# A Data Quality Framework for the European Health Data Space for secondary use

**DOI:** 10.1093/eurpub/ckac129.498

**Published:** 2022-10-25

**Authors:** E Bernal-Delgado, T Engsig-Karup, F Estupiñán-Romero, N Sahlertz Kristiansen, J Bredmose Simonsen

**Affiliations:** 1 Data Sciences for Health Services and Policy, Institute for Health Sciences in Aragon, Zaragoza, Spain; 2 Health Informatics, Aarhus University Hospital, Central Denmark Region, Denmark; 3 Department of Clinical Epidemiology, Aarhus University Hospital, Aarhus, Denmark; 4 CONNECT, Center for Clinical and Genomic Data, Central Denmark Region, Finland

## Abstract

A cornerstone in the development of the European Health Data Space for secondary use of data (EHDS2) is the design, implementation and assessment of a Data Quality Framework (DQF). Consistently, the Joint Action TEHDAS has a dedicated work program where, learning from others’ experiences across Europe and abroad, the work package is building the concepts and methods for such a DQF. The scope of this work program is to provide recommendation to the Member States and the European Commission on the concept of DQF to foster, where (institutions) the DQF should be implemented, when in data life cycle, how should be implemented and by whom. In terms of the concept, the DQF raises the importance of quality assurance procedures at data processor level and the level of quality of the data collections in terms of reliability, relevance, timeliness, coherence, coverage and completeness. When it comes to when along the data life cycle, DQF is expected operate when data needs harmonization at data processor level (ie, the effective application of interoperability standards), in the publication of the data sources (ie providing users knowledge on the provenance of data and the content of data source); or, when data sources have to be integrated and sensitive data pseudonymized (ie, the quality of the linkage and losses after pseudonymisation). Finally, when it comes to the methodology, TEHDAS suggests a three-fold approach - some quality measures in the DQF could be translated into legislation (eg, the requirement of regular auditing for a data processor to be a trusted party in the EDHS2); some could be kept as good-practices (eg, recommendation of archival procedures when a research project finalizes); and, under the assumption of continuous data quality improvement, an assessment, benchmarking and promotion methodology (eg, a grading system at data processor level).

